# Oyster shell-derived nano-hydroxyapatite and proanthocyanidin pretreatment on dentinal tubule occlusion and permeability before and after acid challenge—an in vitro study

**DOI:** 10.1007/s10856-023-06724-4

**Published:** 2023-04-10

**Authors:** Udatha Bhavan Ram, Venkatappan Sujatha, Sampath Vidhya, Raghavan Jayasree, Sekar Mahalaxmi

**Affiliations:** 1 Department of Conservative Dentistry and Endodontics, SRM Institute of Science and Technology, SRM Dental College, Bharathi Salai, Ramapuram, Chennai, 600 089 India; 2 Department of Biomedical Engineering, GKM College of Engineering and Technology, Chennai, 600 063 India

## Abstract

This in vitro study evaluated the dentinal tubule occlusion (TO), depth of penetration (DoP), and dentin permeability (DP) of oyster shell-derived nanohydroxyapatite (os-nHAp) with and without 15% proanthocyanidin (PA) pretreatment. os-nHAp was synthesized via the precipitation method and it was characterized. The morphology and particle size of os-nHAp were analyzed using a high-resolution transmission electron microscope (HRTEM). Cytotoxicity of os-nHAp, PA/os-nHAp, and casein phosphopeptide-amorphous calcium phosphate (CPP-ACP) was assessed by (3-(4,5-dimethythiazol-2-yl) 2,5-diphenyl tetrazolium bromide (MTT) assay using human osteosarcoma (MG-63) cell line. One hundred and ninety-seven dentin discs of 3 mm thickness were prepared from the crown portion of extracted human teeth. The dentinal surfaces of the discs were etched for 2 min with 6% citric acid to simulate dentin hypersensitivity. Five discs were randomly selected and the patency of dentinal tubules was confirmed using a scanning electron microscope (SEM). The remaining 192 discs were divided into four groups (*n* = 48) depending on the type of remineralization as follows: group 1: os-nHAp, group 2: PA/os-nHAp, group 3: CPP-ACP, and group 4: no treatment. The remineralization protocol was followed for 21 days. Out of the 48 dentin discs in each group, 32 discs were used to evaluate dentinal tubule occlusion (TO) and depth of penetration (DoP) using SEM. The remaining 16 discs were subjected to an assessment of dentin permeability (DP) using a hydraulic conductance model. TO, DoP and DP were evaluated after remineralization and acid challenge. Characterization studies confirmed the presence of pure phase apatite. HRTEM confirmed the nanometric particle size of os-nHAp. MTT assay results showed that all the tested materials exhibited >80% cell viability when tested up to a concentration of 100 µg/mL. The results demonstrated a significantly higher mean percentage of TO, DoP, and lesser mean DP after remineralization in groups 1, 2, and 3 (*p* < 0.05). After the acid challenge, group 3 showed a significant reduction in TO and DoP, and increased DP (*p* < 0.05). However, no such changes were observed in groups 1 and 2. Within the limitations of this study, it can be concluded that os-nHAp and PA/os-nHAp could serve as potential and durable therapeutic agents in the treatment of dentin hypersensitivity.

**Graphical Abstract:**

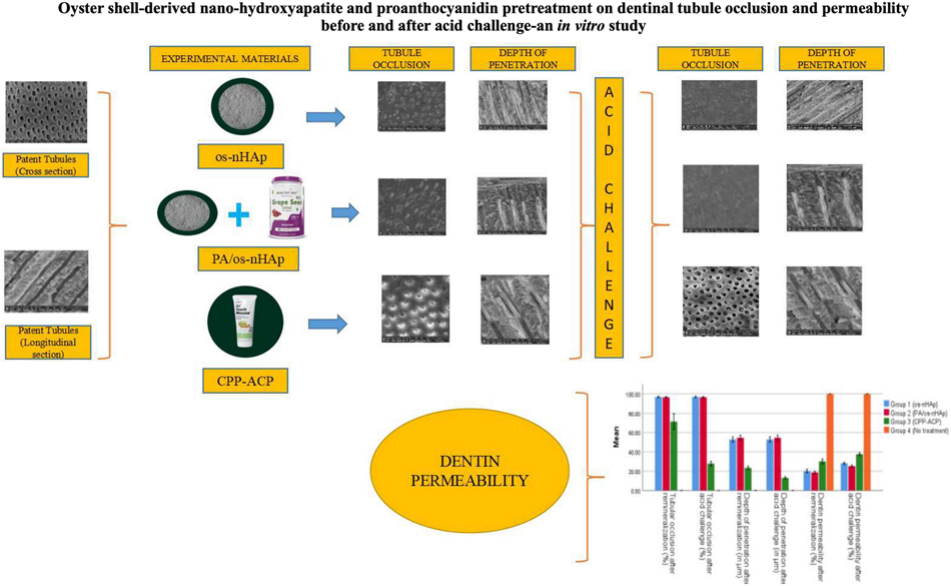

## Introduction

An increase in the life expectancy of human beings and a longer retention period of dentition has made the incidence of dentin hypersensitivity (DH) a frequent finding in clinical practice [1]. DH is defined as “a characteristic pain arising from exposed dentin, in response to thermal, evaporative, tactile, osmotic or chemical stimuli, which cannot be ascribed to any other form of dental pathology” [2]. While the prevalence of DH ranges from 1.3% to 92.1%, a recent systematic review has estimated an average prevalence rate of 33.5% [3]. According to Brännström’s hypothesis, DH is caused by an increased multidirectional fluid flow within the dentinal tubules (DTs), which is triggered by an external stimulus. The fluid movement activates the nerve terminals at the pulp-dentin interface resulting in sharp and shooting pain [4]. This finding led to the treatment of DH with two different strategies namely, nerve desensitization using potassium salts and limiting intratubular fluid movement with agents that could produce tubule occlusion (TO) [1, 5]. Suge et al. hypothesized that the goal of a desensitizing agent is to provide fast and long-lasting pain relief by enabling surface as well as intratubular occlusion [6]. Considering these requirements, materials with a bioactive potential that could instantly block the exposed dentinal tubules and sustain the TO over long term are being widely researched.

Hydroxyapatite (HAp) is one such bioactive calcium phosphate compound, used in the treatment of DH. The particle size of HAp plays a key role in harnessing its advantages in biological applications. Compared to microscale counterparts, nanoscale dimensions could result in distinct activities by the particles. Their large surface area and small size enhance the hydration of the material, resulting in improved physical and chemical characteristics [7].

The source of nano-hydroxyapatite (nHAp) could be natural or synthetic. With the increase in demand for environment-friendly techniques, synthesizing nHAp from calcium-rich natural sources has proven to be a viable and more economical option [8]. The various natural sources of calcium include animal bone [9], avian eggshells [8], shells of oysters [10], and many others [8]. As aquatic animals form a vital component of the food chain, seafood consumption by humans results in an enormous generation of this shell waste [11]. Rujitanapanich et al. demonstrated the synthesis of nHAp from oyster shells (OS) using the precipitation method [10]. OS is composed of approximately 96% calcium carbonate, trace amounts of oxides of Na, Mg, Si, Al, and moisture [10, 12]. Studies have shown that chicken eggshell-derived nHAp occluded patent DTs effectively and suggested that it could be a potential therapeutic agent for DH [13, 14]. But, the remineralizing potential of nHAp derived from OS (os-nHAp) is yet to be tapped in dentistry.

Owing to the loss of enamel and long-standing exposure of denuded dentin to harsh oral conditions, the integrity of collagen is questionable in DH. Strategies aimed at treating DH should improve the mechanical and chemical stability of the demineralized dentin so that it serves as a scaffold for mineral deposition [15]. Intermolecular crosslinking is vital for the stability of dentin collagen. Various exogenous collagen crosslinkers (CCLs) have been extensively researched and evidenced to preserve the structural integrity and functionality of collagen. One such CCL is proanthocyanidin (PA), a class of polyphenolic compounds found in various natural products such as seeds, nuts, fruits, vegetables and barks of trees. Grape seed extract used in the present study is a rich source of PA and has a proven capability of strengthening collagen-based tissues by increasing collagen cross-linking [16]. It has been shown that in the presence of a calcium source, 15% PA is capable of facilitating the deposition of HAp onto the demineralized dentin [17]. Casein phosphopeptide-amorphous calcium phosphate (CPP-ACP) serves as a calcium-phosphate reservoir around the teeth and has been used as a therapeutic agent in the treatment of DH due to its ability to block open dentinal tubules [18, 19]. Hence the aim of this in vitro study was to evaluate the effect of surface application of os-nHAp with and without 15% PA pretreatment in comparison with CPP-ACP on dentin tubule occlusion and intratubular depth of mineralized precipitate penetration. The dentin permeability was measured using hydraulic conductance before and after an acid challenge after 21 days. The null hypothesis was that os-nHAp with and without PA pretreatment will not have any effect on the above-mentioned parameters.

## Materials and methods

### Synthesis of nHAp from oyster shell

The precipitation method was adopted to synthesize nHAp from oyster shells [10]. The chemicals used in this synthesis were of analytical grade and were purchased from Merck India, Mumbai, India. Oyster shells collected from a local farm (Supreme seafood, Chennai, India) were washed, dried in an oven at 300 °C for 1 h, powdered and then sieved with a 50-mesh sieve. The chemical reactions involved in the synthesis of nHAP are given below.

$$CaCO_3 \to CaO + CO_2 \uparrow$$

$$CaO + 2HNO_3 \to Ca\left( {NO_3} \right)_2 + H_2O$$

$$10Ca\left( {NO_3} \right)_2 + 6\left( {NH_4} \right)2HPO_4 + 8NH_4OH \to Ca_{10}\left( {PO_4} \right)_6\left( {OH} \right)_2 + 20NH_4NO_3 + 6H_2O$$

The oyster shell powder was calcined in an electrical muffle furnace at a rate of 10 °C/min and maintained at a temperature of 1200 °C for 2 h to form calcium oxide (CaO). 3 g of CaO powder dissolved in distilled water was then converted to calcium nitrate (Ca(NO_3_)_2_) solution by the addition of 25% nitric acid under constant stirring. Diammonium hydrogen phosphate ((NH_4_)_2_HPO_4_) solution (12.73 g in 30 mL distilled water) was then added to calcium nitrate solution under constant stirring at room temperature. Liquid ammonia was added in drops to maintain the pH of the reaction between 8 and 10. The solution was continuously stirred for 1 h to achieve a homogeneous solution, following which it was subjected to aging for 24 h and the precipitated HAp was filtered from the solution using Whatman filter paper. The filtered precipitate was dried in a hot air oven at 120 °C for 2 h, and then calcined at 900 °C for 2 h to obtain nHAp.

### Characterization of the synthesized os-nHAp powder

The crystalline nature and lattice parameters were determined by X-ray diffraction (XRD, Rigaku Ultima IV X-ray diffractometer, Tokyo, Japan) using Cu Kα radiation (*λ* = 0.154 nm). The data were analyzed in the 2*θ* range with a scanning step of 2^o^ per minute. Using Fourier transform infrared spectrometer (FTIR, Perkin Elmer spectrum RX1 spectrometer, Chennai, India), the infrared spectrum was recorded in the wavelength ranging from 400 to 4000 cm^−1^ using the KBr pellets technique. The morphology and particle size were characterized using a high-resolution transmission electron microscope (HRTEM) (JEOL-JEM-3010, JEOL USA Inc, MA, USA) operating at an accelerating voltage of 200 kV and at a magnification range of 40,000×–50,000×. The particles were deposited on a 300-mesh carbon-coated copper grid. A Gatan 794 multiscan CCD camera was used for image acquisition and the particle size was analyzed using ImageJ software.

### In vitro cytotoxicity assay

#### Reagents used

Dulbecco’s modified Eagle’s medium (DMEM), phosphate-buffered saline (PBS) and fetal bovine serum (FBS) were purchased from Gibco, Fisher Scientific Company, Ottawa, Canada. Dimethyl sulfoxide (DMSO) and (3-(4,5-dimethythiazol-2-yl) 2,5-diphenyl tetrazolium bromide (MTT) were purchased from Sigma-Aldrich Chemicals Pvt. Ltd., Bangalore, India. All other chemicals used were extra pure molecular grade and were purchased from Sisco Research Laboratories Pvt. Ltd., Mumbai, India.

#### Cell culture

The human osteosarcoma cell line (MG-63) was obtained from the National Center for Cell Science (NCCS, Pune, India). The cells were grown in T25 culture flasks containing DMEM supplemented with 10% FBS and 1% antibiotics. Cells were maintained at 37 °C in a humidified atmosphere containing 5% CO_2_. Upon reaching confluency, the cells were trypsinized and passaged.

#### MTT assay

MG-63 cell lines were plated separately in 96-well plates with a concentration of 5 × 10^3^ cells/well in DMEM supplemented with 10% FBS and 1% antibiotic solution in CO_2_ incubator at 37 °C with 5% CO_2_. The cells were washed with 100 μL of 1% PBS, and then were treated with different concentrations of samples (25 to 100 μL/mL) and incubated in CO_2_ incubator at 37 °C with 5% CO_2_ for 24 h. The control cells received media alone. The medium was aspirated from cells at the end of the treatment period. 0.5 mg/mL MTT prepared in 1% PBS was added and incubated at 37 °C for 4 h in CO_2_ incubator. After the incubation period, the medium containing MTT was discarded from the cells and washed using 100 μL of PBS. The formed crystals were dissolved with 100 μL of DMSO and thoroughly mixed. The absorbance was measured at 570 nm using a microplate reader. The percentage of cell viability was measured using the formula: Cell viability = (Optical density (OD) of treated cells/OD of control cells) × 100. The experiment was done in triplicate and the mean percentage of cell viability was calculated.

### Preparation of tooth samples

**Fig. 1:**
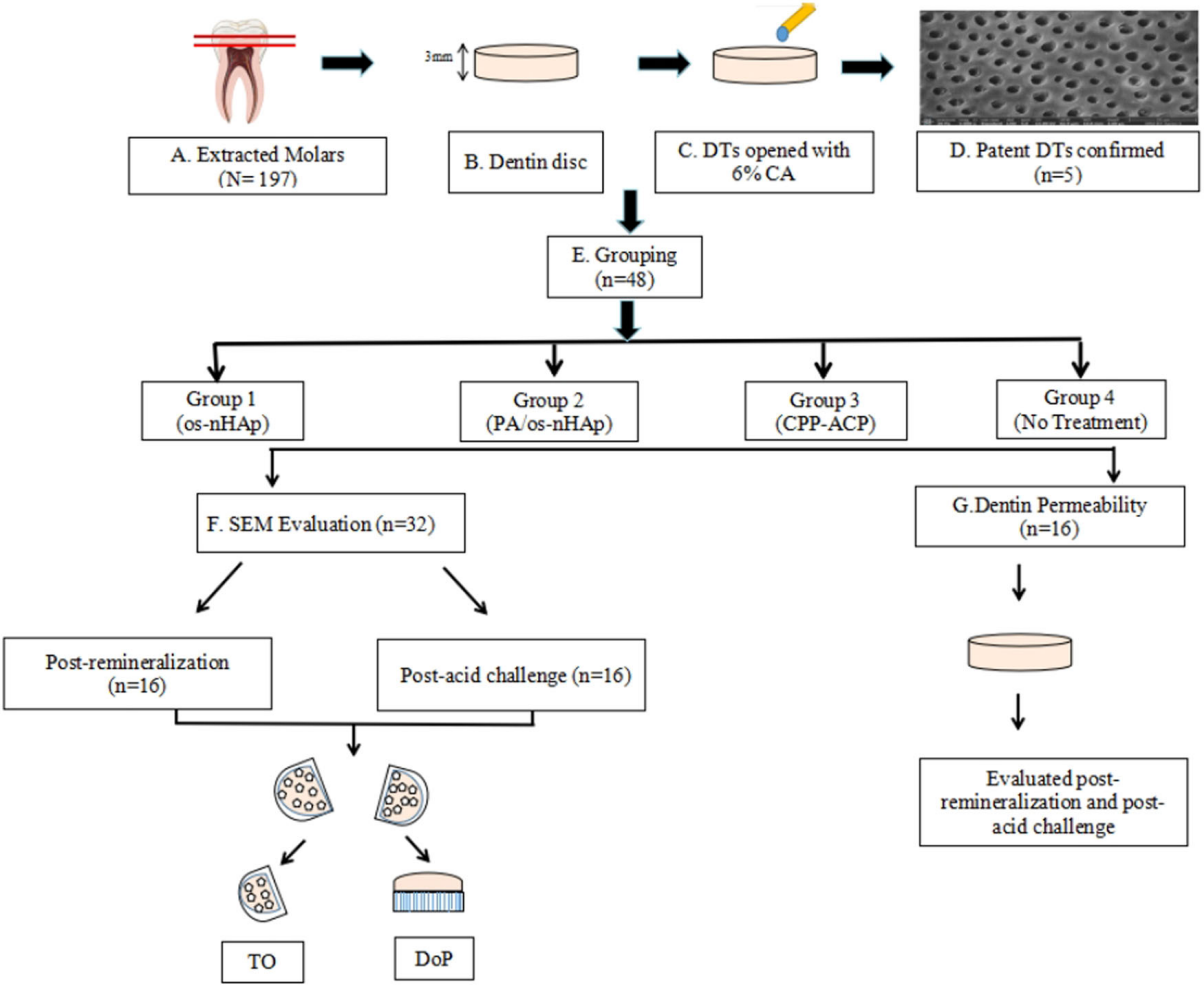
Schematic representation of methodology. DTs-dentinal tubules, CA-6% citric acid, TO-tubule occlusion, DoP-depth of penetration

The study was conducted at SRM Institute of Science and Technology. The study protocol was duly submitted to the Institutional Review Board of SRM Dental College and approval was obtained (SRMDC/IRB/2019/MDS/No.306). The ethical standards outlined in the 1964 Declaration of Helsinki and its later amendments were followed in the collection of tooth samples. The teeth were extracted after obtaining written informed consent from patients. The schematic representation of the methodology is given in Fig. 1. 197 human maxillary or mandibular molars free of caries, cracks and developmental anomalies extracted for periodontal reasons were collected and stored in 0.1% thymol solution until use. The crowns were secured by decoronating the teeth at the level of the cemento-enamel junction using a diamond disc (GDC, Hoshiarpur, India) under copious water cooling. A 3 mm thick dentin disc was obtained from each crown by making two horizontal cuts parallel to each other and perpendicular to the long axis of the tooth using a water-cooled diamond disc. The occlusal surface of the discs was polished with ascending grades of silicon carbide paper (Carborundum Universal Ltd., Chennai, India), starting from 600 to 1000 grit. The samples were ultrasonicated in distilled water for 30 min to remove the smear layer formed during polishing.

In order to mimic the clinical condition of DH, the exposed dentinal surfaces were treated with 6% citric acid (CA) (Labogens, Ahmedabad, India) for 2 min followed by thorough rinsing with distilled water. Five discs were randomly selected and the uniformity of demineralization was confirmed under SEM (FEI Quanta 200 FEG, FEI Co., Hillsboro, USA). Based on the remineralizing treatment protocol, the remaining 192 discs were randomly allotted to four groups of 48 discs each as follows:

### Group 1: os-nHAp

1.8 g of os-nHAp was measured using an electronic weighing machine (K. Roy & Co., Kolkata, India), dispensed into 0.1 L of 2% acetic acid and stirred for 30 s to obtain a slurry of os-nHAp. Two coats of this slurry were applied on the exposed dentinal surface using an applicator tip, left in place for 7 min and rinsed off with distilled water.

### Group 2: PA/os-nHAp

15% PA solution was prepared by dissolving 15 g of grape seed extract (GSE) powder (Healthy Hey foods LLP, Mumbai, India) in 100 mL of distilled water. Two consecutive coats of 15% PA solution were applied on the dentin disc and left in place for 2 min. The PA solution was thoroughly rinsed off with distilled water followed by the application of os-nHAp slurry as mentioned under group 1.

### Group 3: CPP-ACP

CPP-ACP paste (Tooth Mousse, GC America Inc., USA) was applied on the dentin disc using an applicator tip, left in contact with the disc for 5 min and rinsed off with distilled water.

Samples in groups 1 to 3 were stored in artificial saliva [20] at 37 °C for the rest of the day after the remineralizing treatment. This 24-h protocol was followed for the next 21 days.

### Group 4: No treatment

The dentin discs did not receive any kind of remineralizing treatment and were stored in artificial saliva at 37 °C for 21 days, with the solution being renewed once every week.

Out of the 48 dentin discs in each group, 16 discs were selected randomly and used to evaluate post-treatment tubule occlusion under SEM. Another 16 discs from each group were subjected to a post-treatment acid challenge (6% citric acid solution for 2 min) prior to the evaluation of tubule occlusion characteristics under SEM. Dentin permeability was evaluated in the remaining 16 discs in each group before and after the acid challenge using a custom-made device.

### Sample preparation for SEM analysis

A groove was made on the dentin discs using a diamond saw and the discs were split into two halves using a chisel and mallet. The split discs were ultrasonicated in distilled water for 10 minutes to remove the debris. In one half of each disc, the occlusal surface was viewed to determine the extent of tubule occlusion. In the other half, the longitudinal section was viewed to determine the depth of intratubular precipitate penetration. Both halves were immersed in 1% glutaraldehyde in PBS for 4 h at 4 °C. The samples were rinsed with PBS, subjected to graded ethanol dehydration and examined under SEM for assessing the percentage of TO and the DoP into the DTs. The elemental surface composition of the treated dentin samples was analyzed using SEM-EDX.

### Evaluation of TO

The ratio of occluded tubules was calculated from the micrographs, using an image analysis software (Adobe photoshop software, V 12.4, SJ, USA) by dividing the area of partially or fully occluded tubules by the total tubules area using 5000x magnification images [21]. This was calculated using micrographs taken from three different regions on the same specimen and average value for the 16 specimens was taken.

### Evaluation of DoP

With the help of an in-built measuring tool in the SEM, the depth of intratubular precipitate penetration was measured along the length of the DTs, from the coronal surface of the formed precipitate to the point in the DTs where the precipitate formation was no longer discernible. This was calculated for 20 tubules in each sample and the average value was taken as the DoP for that sample [13].

### Evaluation of dentin permeability (DP)

**Fig. 2:**
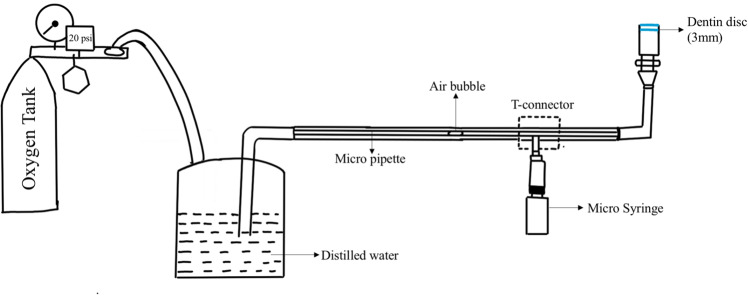
Schematic representation of the hydraulic conductance model

A schematic representation of the hydraulic conductance model used in this study is shown in Fig. 2. The pulpal side of the disc was placed inside a plastic holder, connected to a stainless steel tube (18 gauge). Cyanoacrylate glue was applied circumferentially between the plastic tube and the disc to obtain an airtight seal. Throughout the experiment, a digital air pressure regulator was used to maintain a constant pressure of 20 psi. All pipettes, plastic tubes and syringes were filled with distilled water. An air bubble was introduced into the micropipette using a microsyringe. The air bubble movement in the micropipette was converted into hydraulic conductance (Lp) [L/(cm^2^ min cmH_2_O)] using the following equation: Lp = *V*/*PS*, where *V*, *P*, and *S* represented volume flow (L/min), water pressure (20 cm H_2_O) and exposed dentin surface area (cm^2^) respectively. Following simulation of hypersensitive dentin, samples showed an increased hydraulic conductance representing maximum permeability, which was considered as baseline. The reduction in dentin permeability following remineralization was expressed as a percentage of the maximum permeability.

### Statistical analysis

Kruskal–Wallis test was used for inter-group comparisons. Intra-group comparisons were done using Wilcoxon signed-rank test. Spearman’s correlation was done to assess the strength of association of the variables. The significance level was set at 0.05.

## Results

### Characterization of synthesized os-nHAp

#### XRD analysis

**Fig. 3:**
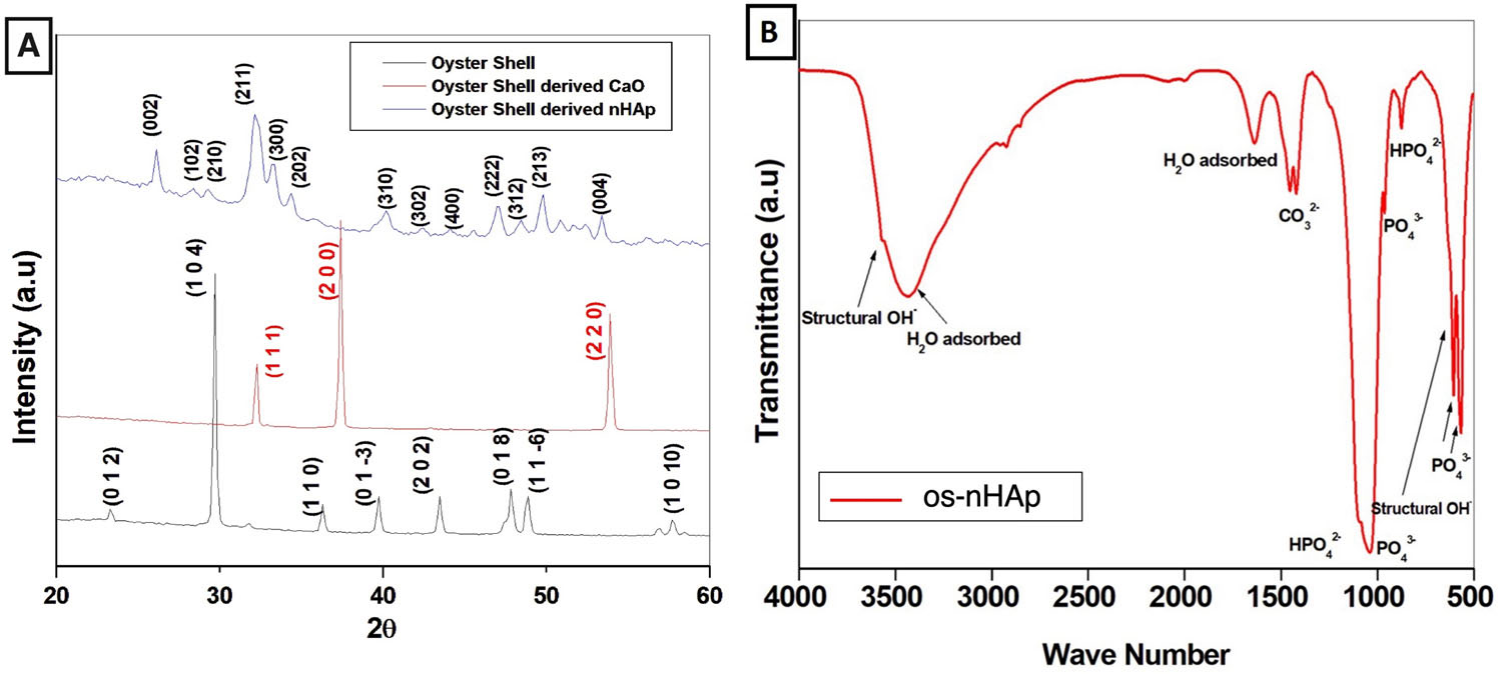
Characterization of os-nHAp powder. **A** XRD spectra **B** FTIR spectra

The XRD pattern of the cleaned oyster shells, oyster shell derived CaO and as-synthesized os-nHAp is shown in Fig. 3A. The peaks identified in oyster shell correspond to the JCPD file 5-0586 and is hence confirmed as calcite. The XRD pattern of oyster shell heated to 900 °C corresponds to JCPD file 37-1497 and is hence confirmed as CaO. The os-nHAp showed the characteristic peak of HAp (JCPDS 9-432). The absence of other phases in os-nHAp confirmed the formation of pure monophasic apatite. The average crystallite size of the synthesized os-nHAp was calculated from the broadening of the peak at 26° corresponding to (0 0 2) reflection using Scherrer’s formula [22]. The cell parameters of os-nHAp were calculated by the least-squares fit method using the program “UnitCellWin” [23] and were found to be: *a* = 9.311 Å and *c* = 6.321 ÅA^o^. The cell parameters were similar to the reported values [22].

#### FTIR analysis

Figure 3B shows the typical FTIR spectra of os-nHAp. The vibration bands at 570, 602, 960, and 1030 cm^−1^ indicate the presence of PO_3_^4−^ groups. The bands at 623 and 3575 cm^−1^ correspond to structural OH^−^. The presence of HPO_2_^4−^ at 875 cm^−1^ indicates that the synthesized os-nHAp was calcium deficient in nature [22].

#### Particle size analysis

**Fig. 4:**
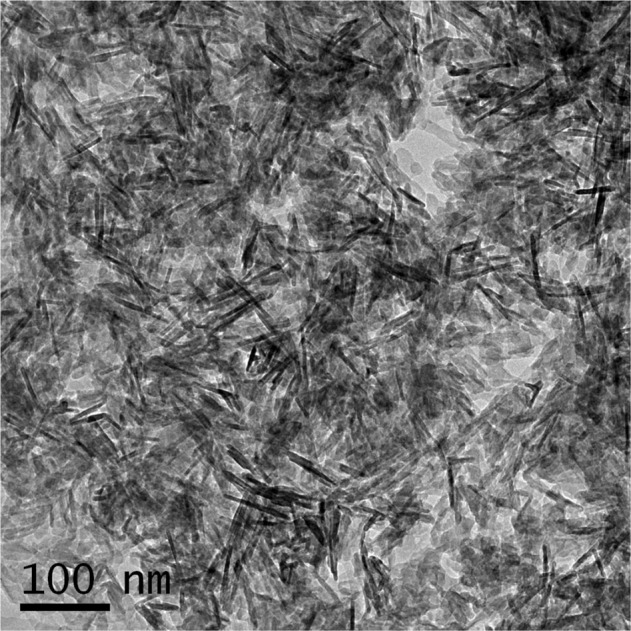
HRTEM micrograph showing morphology and size of os-nHAp particles

HRTEM micrograph showing morphology and size of os-nHAp particles is given in Fig. 4. Rod-shaped os-nHAp particles with an average crystallite size of 31.93 ± 2.1 nm were evidenced.

#### In vitro cytotoxicity assay

**Fig. 5:**
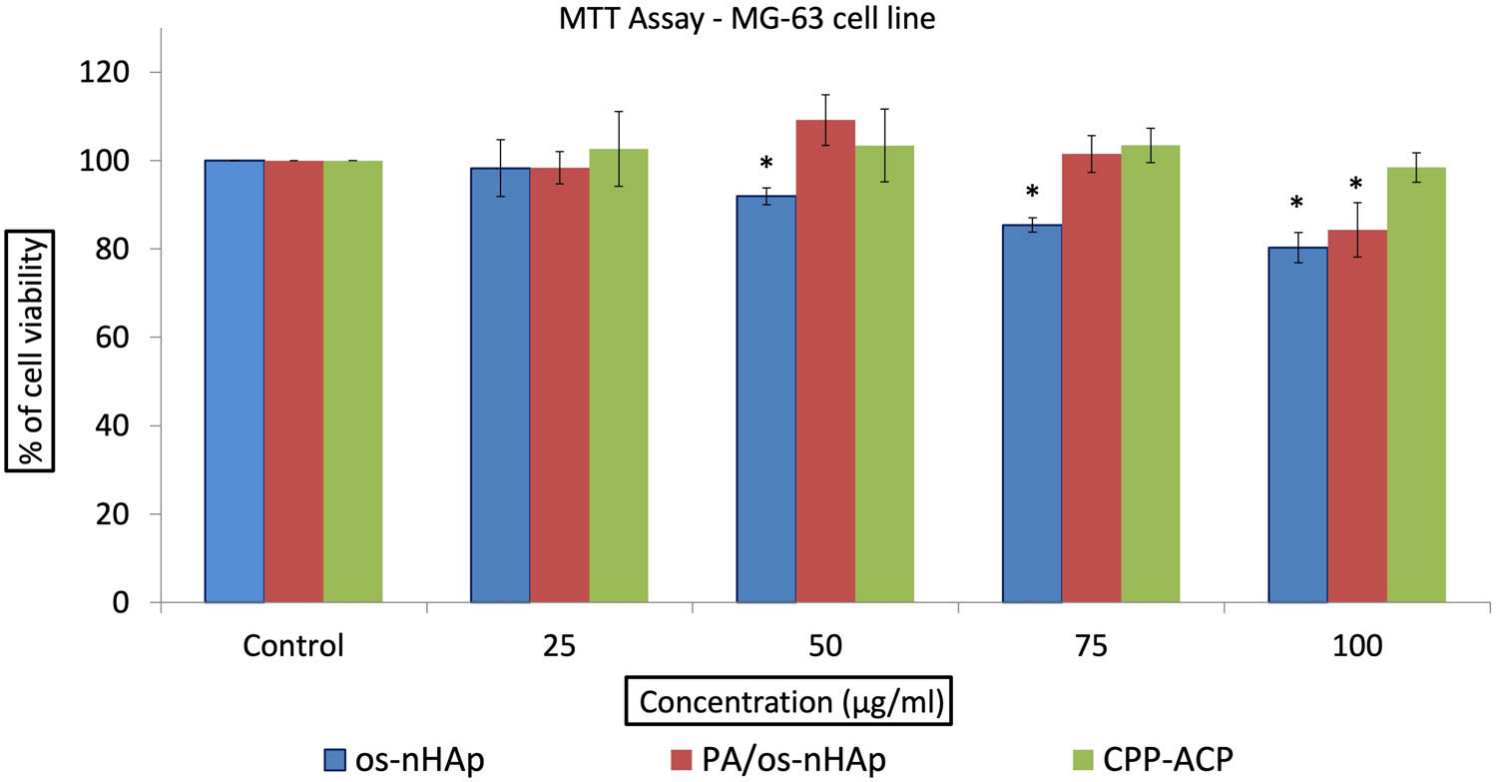
The mean (±S.D) percentage of cell viability of os-nHAp, PA/os-nHAp, and CPP-ACP. *denotes a significant difference compared to the control (*p* < 0.05)

A graphical representation showing the mean (±S.D) percentage of cell viability of os-nHAp, PA/os-nHAp, and CPP-ACP is given in Fig. 5. Cells which received media alone (control) showed 100% cell viability. Os-nHAp and PA/os-nHAp showed 80% cell viability up to a concentration of 100 µg/mL and differed significantly from the control (*p* < 0.05) whereas, CPP-ACP did not significantly differ from the control (*p* > 0.05).

#### SEM-EDX analysis of remineralized dentin

**Fig. 6:**
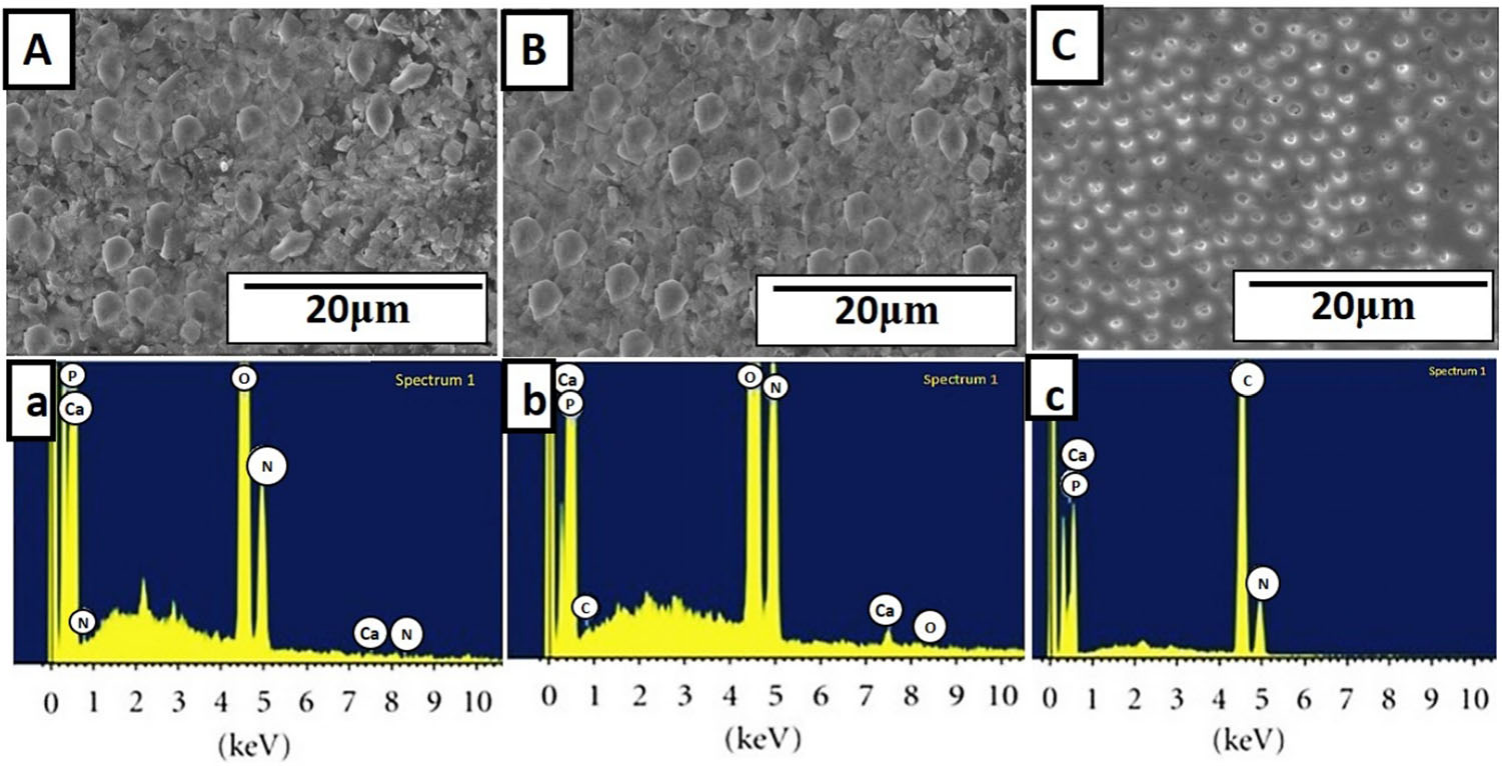
SEM images of remineralized dentin surface (upper case letters) and their corresponding EDX analysis (lower case letters) of os-nHAp (**A**,a), PA/os-nHAp (**B**,b) and CPP-ACP (**C**,c) groups

SEM images of remineralized dentin surface showed deposition of minerals in os-nHAp (Fig. 6A), PA/os-nHAp (Fig. 6B), and CPP-ACP (Fig. 6C) groups. EDX analysis of the remineralized dentin surface confirms the presence of Ca and P in the ratio of 1.67, 1.68, and 1.67 in os-nHAp (Fig. 6a), PA/os-nHAp (Fig. 6b), and CPP-ACP (Fig. 6c), respectively.

#### Qualitative assessment of TO and DoP

**Fig. 7:**
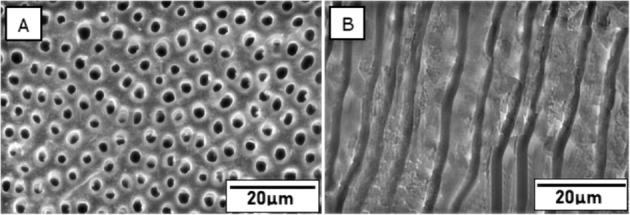
SEM image of citric acid treated dentin confirming tubule patency in cross- section (**A**) and longitudinal section (**B**)

**Fig. 8:**
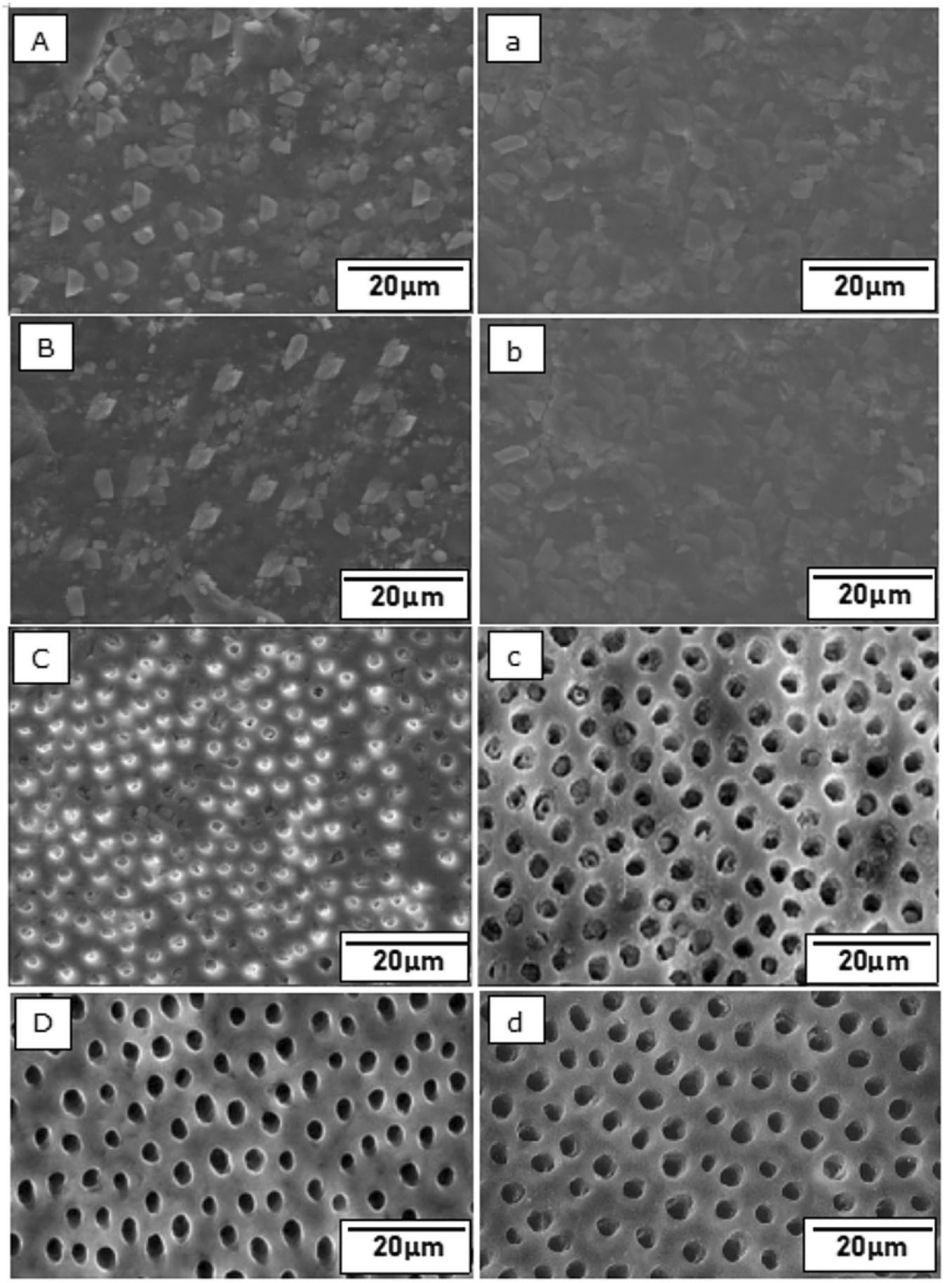
SEM micrographs showing tubular occlusion post-remineralization (upper case letters) and post-acid challenge (lower case letters) in os-nHAp (**A**,a), PA/os-nHAp (**B**,b), CPP-ACP (**C**,c) and no treatment (**D**,d) groups

**Fig. 9:**
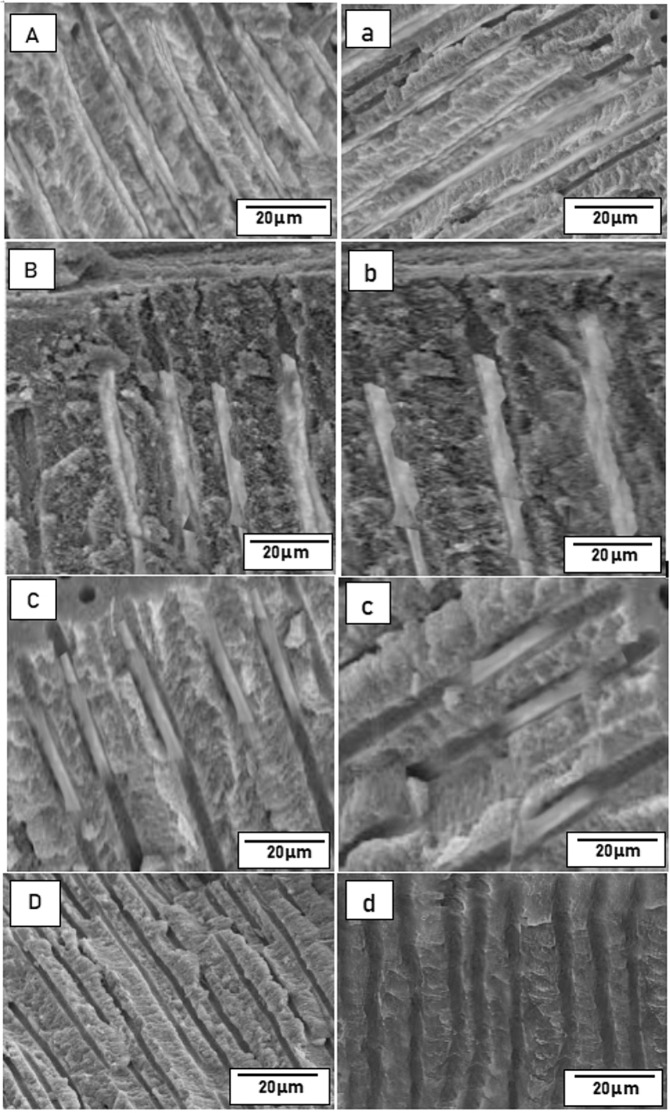
SEM micrographs showing depth of precipitate penetration post-remineralization (upper case letters) and post-acid challenge (lower case letters) in os-nHAp (**A**,a), PA/os-nHAp (**B**,b), CPP-ACP (**C**,c) and no treatment (**D**,d) groups

The application of 6% citric acid for 2 min resulted in open dentinal tubules uniformly throughout the sample (Fig. 7). SEM micrographs showing tubular occlusion and intratubular penetration following remineralization and post-acid challenge in all the groups are given in Figs. 8 and 9, respectively. Group 1 (os-nHAp) and group 2 (PA/os-nHAp) showed occlusion of a predominantly higher number of DTs. Increased surface deposition of minerals was observed in PA-treated samples. Group 3 (CPP-ACP) showed occlusion of the DTs, however, the plugs were loosely attached to the tubular orifices. Several open DTs were also seen in this group. Cross-sectioned samples revealed intratubular precipitate plugs in groups 1-3. Patent DTs with no visible TO or intratubular plug were evidenced in group 4 (no treatment).

#### Quantitative assessment of TO and DoP

The mean percentage of tubular occlusion (TO) after remineralization in group 1 (os-nHAp), group 2 (PA/os-nHAp), and group 3 (CPP-ACP) were 96.88, 96.57, and 71.30, respectively. The mean DoP (in µm) into the DTs after remineralization in os-nHAp, PA/os-nHAp and CPP-ACP were 52.82, 54.62, and 23.47, respectively. Group 4 (no treatment) did not show any TO and intratubular precipitate and hence was excluded from further interpretation. Groups 1 and 2 showed significantly higher mean percentage of TO and increased mean DoP compared to group 3 (*p* < 0.05). No significant difference was observed among groups 1 and 2 in TO (*p* = 1.000) and DoP (*p* = 0.844).

#### Dentin permeability

The mean dentin permeability (in %) after remineralization in group 1 (os-nHAp), group 2 (PA/os-nHAp), group 3 (CPP-ACP), and group 4 (no treatment) were 20.13, 18.65, 30.10, and 100, respectively. Groups 1 and 2 showed significantly lesser DP compared to group 3 (*p* < 0.05). No significant difference was observed among groups 1 and 2 (*p* = 0.475). DP of group 3 following remineralization was significantly lesser than the no-treatment group (*p* < 0.05).

#### Acid challenge

Following the acid challenge with 6% CA, the dissolution of surface minerals was seen in groups 1 and 2. But, tubule orifices remained occluded and reopening of DTs was not observed. Intact intra-tubular plugs were still evident in both groups. In group 3, resurfacing of patent DTs, widening of tubular orifices along with a visible loss of TO and intratubular plugs could be noticed. The sparse mineralized surface layer that was seen in CPP-ACP treated samples post-remineralization was dissolved completely by the action of the acid. Surface erosion of the dentin was also visibly present. In cross-section, it could be observed that fewer tubules were able to retain a partial occluding plug much farther from the surface of DTs.

**Fig. 10:**
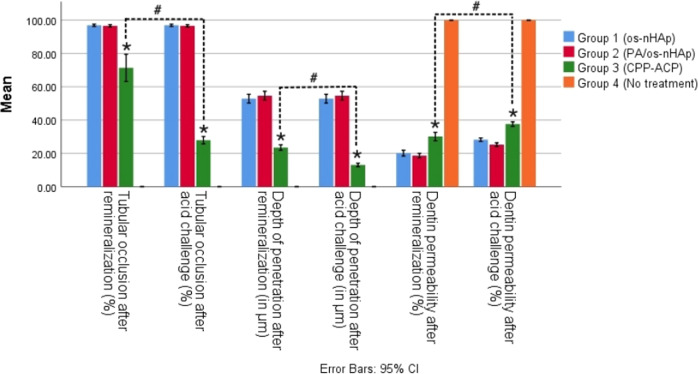
The means of TO, DoP, and DP in all the groups at both evaluation periods. *denotes a significant difference between CPP-ACP and the nHAp groups under each parameter. ^#^denotes a significant difference between post-remineralization and post-acid challenge values in the CPP-ACP group under each parameter

CPP-ACP showed a loss of 60.37% of TO and a loss of 44.75% of intratubular plugs from the DTs and a 22.69% increase in DP following acid challenge which was significantly higher than os-nHAp and PA/os-nHAp. Under TO (*p* = 1.000), DoP (*p* = 0.765) and DP (*p* = 0.077), no significant difference could be observed between the os-nHAp and PA/os-nHAp groups. Figure 10 shows the graphical representation of the percentage of TO, mean DoP and mean DP of all the groups at both the evaluation periods. Spearman’s correlation coefficient showed a strong negative correlation in the post-acid challenge values of DoP and DP in the CPP-ACP group (−0.565, *p* < 0.05).

## Discussion

The objectives of the study were to synthesize nHAp using oyster shells and to assess its effectiveness with and without PA pretreatment in occluding tubules in dentin with simulated DH. The integrity of the occluded precipitates to acid challenge and hydraulic conductance were also assessed. The characterization studies confirmed the formation of pure phase nHAp nanoparticles from oyster shells through the precipitation method. The propensity of a biomaterial to cause acute toxicity is determined by cytotoxicity tests [24]. In the current study, human osteosarcoma MG-63 cells were exposed to os-nHAp, PA/os-nHAp, and CPP-ACP. The materials were biocompatible within the range of concentrations tested.

In the current study, CPP-ACP achieved an appreciable amount of TO and formed precipitate plugs into the DTs compared to no treatment group, which is in accordance with the findings of Ghafournia et al. [18]. CPP forms nanoclusters with ACP, which can maintain the supersaturation of ions in the saliva thereby resulting in mineralization. The precipitate formed is predominantly found to be dicalcium phosphate dihydrate (CaHPO_4_ 2H_2_O) [19]. Kijsamanmith et al. evaluated the TO by CPP-ACP and showed that it was able to only partially cover the DTs leaving most of the tubules patent [25]. A similar observation could be made in the present study also, where peripheral voids surrounding the tubule plugs could be seen, showing that a complete juxtaposition of the intratubular precipitate with the peritubular dentin had not occurred. Other than a discrete deposition of microscopic particles, no other visible mineralized precipitate could be appreciated on the intertubular dentin surface of the samples in this group. The inability to completely occlude the DTs could be attributed to the slow release of calcium and phosphate ions and their precipitation. The neutral pH of CPP-ACP is also cited as responsible for its slower ionic dissociation [25].

In contrast to CPP-ACP, os-nHAp, and PA/os-nHAp were able to achieve almost complete TO (>96%) and also formed a dense mineralized surface layer. Hence, the null hypothesis is rejected. Very few visible patent DTs and partially occluded tubules could be seen in these samples. This could be attributed to the release of calcium and phosphate in high concentrations, which strongly bind to the dentin and transform into HAp leading to an effective TO [26]. Numerous studies support the TO potential of nHAp [27, 28]. Luong et al. experimented with a novel nHAp- containing desensitizer and inferred that nHAp deposited into the patent DTs, sealing them effectively and also formed a thick mineralized layer on demineralized dentin [26]. In an environment supersaturated with HAp and abundant calcium ions, nHAp acts as nucleation sites. According to Huang et al., this supersaturated state is dependent on the availability of calcium ions, which are released in higher proportions from nHAp than its micro-sized counterpart [29]. Amaechi et al. evidenced the superior TO potential of nHAp pastes and made a vital observation that the longer the period of desensitizer application, the greater the possibility of achieving complete TO and mineral precipitate layer formation on the surface [30]. These findings reinforce the importance of long-term remineralizing therapy in the successful management of DH. Hence, a 3-week remineralizing regimen was followed in the present study. Various studies in the literature evaluated the TO potential of nHAp, in comparison to CPP-ACP, the results of which are in accordance with the present study [31, 32]. Contrary results were obtained by Mathew et al. in a similar study on cervical dentin of extracted primary molars [33]. The use of microsized HAp and the different substrates tested could have contributed to the difference in their results.

The significantly greater DoP observed with both the os-nHAp groups compared to CPP-ACP could be attributed to the nanometric size of the synthesized nHAp particles, which are much smaller than the DT diameter (1.2–2.5 μm in mid-coronal dentin) [34], facilitating deeper penetration into the long and narrow DTs. Complete integration of the mineralized plug with the peritubular dentin could be appreciated in os-nHAp and PA/os-nHAp treated samples. This could be attributed to the acidic pH of the os-nHAp slurry (2.5–3), which might have exerted a mild etching effect on the peritubular dentin resulting in the release and reprecipitation of calcium ions. This could have further been potentiated in the presence of a remineralizing agent leading to a deeper and intact sealing of the DTs. In addition, the subtle roughening of peritubular dentin caused by the acidic remineralizing agent might have enhanced the micromechanical bonding of the mineralized precipitate to the dentin. These results can be related to the findings of Yu et al. who advocated the use of an acidic remineralizing agent [35]. However, a previous study had shown that although avian chicken eggshell-derived nHAp exhibited similar DP as CPP-ACP, its tubule occluding potential and acid resistance were significantly superior to CPP-ACP. However, the authors hypothesized that nHAp could have only superficially occluded the DTs [14]. In the present study, it is evident that nHAp had formed deeper plugs into the DTs. Future studies should be done to compare and evaluate the tubule occluding capability of nHAp obtained from different natural sources.

The occlusion of DTs with remineralizing agents must withstand the forces of abrasion and pH changes to have a long-term effect on DH. The integrity of TO post-remineralization can be evaluated using an acid challenge test or the DP test [25]. CA was used for the acid challenge in the present study as it is a common ingredient of fruits, fruit juices, and other beverages consumed [14]. Dias da Cruz et al. used 6% CA for 2 min to study the resistance offered by desensitized dentin to acid [36]. The same protocol is followed in the present study.

Following the acid challenge, the CPP-ACP group exhibited a significant reduction in the percentage of TO, suggestive of the rapid solubility of the deposited calcium phosphate crystals. This finding reiterates the fact that CPP-ACP is a poor contender against acid challenge, which could be commonly encountered in the day-to-day activity of the patients thereby compromising dentin protection and pain relief from DH. The results are in accordance with the study by Wang et al. and Berg et al. who observed a complete loss of the plugs occluding the DTs in CPP-ACP treated samples after acid challenge [37, 38].

Exposure of os-nHAp and PA/os-nHAp remineralized samples to CA revealed mild erosive changes on the surface mineralized layer, leaving the tubular plugs intact (>96%). Previous studies also made similar observations with nHAp-containing desensitizers and found them to be more resistant to dissolution by acid [38, 39]. This could be attributed to the mineralized calcium phosphate formed on the surface, which is less soluble at low pH. Further, it was observed that there was no loss of precipitate present within the DTs following the acid challenge. The acid-resistant surface crystalline layer that was formed in the os-nHAp and PA/os-nHAp treated samples would have prevented the acid from reaching the DT orifice resulting in more retention of these precipitates within the DTs.

EDX analysis of PA-pretreated os-nHAp samples showed that the mineralized layer formed on these samples was predominantly composed of high concentrations of calcium and phosphate. The Ca/P ratio of ~1.67 indicates that the deposited mineral phase is a stoichiometric HAp [22]. Previous studies have proposed PA as a potential remineralization-promoting agent [16, 17]. PA is capable of forming insoluble complexes in combination with remineralizing agents. The complexes formed were resistant to dissolution even at an acidic pH of 2 [16]. A similar finding was observed in the current study. Two-fold reasoning was put forth to explain the mechanism of mineralization by GSE. Primarily, PA is capable of attracting calcium from the remineralizing solution leading to the deposition of a mineralized layer on the surface of the PA-pretreated substrate, correlating with the present study. Secondly, an intact collagen scaffold is necessary in order to receive the minerals, which is assured by the action of PA. PA interacts with the proteins present in the organic portion of the collagen by forming ionic, covalent, or hydrogen bonds or by way of hydrophobic interactions. This increases the formation of cross-links between the collagen molecules, stabilizing the demineralized dentinal collagen structure and preparing it to be a suitable substrate for mineral deposition during remineralization [16].

Studies have shown that following an acid challenge, hydraulic conductance of dentin and DP increased by 32–46 times and 96.8%, respectively [40, 41]. Hence, an assessment of DP could validate the TO potential of desensitizing agents. A significant reduction was seen with CPP-ACP, while DP remained unaltered after the acid challenge in the os-nHAp and PA/os-nHAp groups. The high solubility of the sparse mineralized layer and shallow intratubular plugs formed by CPP-ACP could be the reasons behind the increased DP seen in this group, as proven in earlier studies [25, 38, 42]. The surface mineralized layer and deeper intratubular plugs, well-integrated with the DT wall, could be the reasons for the superior performance of the os-nHAp groups in the present study, the results of which are in accordance with previous reports [41, 43]. The correlation of results of TO, DoP, and DP reinforces the fact that these parameters are closely related according to the hydrodynamic hypothesis.

This in vitro study demonstrated the desensitizing potential of an indigenously developed nHAp using oyster shells as a natural source. The experimental material showed remarkable TO potential and penetrated deeper into the DTs. In addition, this dentinal sealing effect was resistant to acidic challenges. These findings are suggestive that os-nHAp could serve as a potential dentin-desensitizing agent. In addition, recycling oyster shell waste into biogenic substances could serve as a viable option to increase biomaterial productivity as well as to reduce the burden of waste disposal on the environment [11].

The limitations associated with the present study include the varying consistencies of the control (paste) and experimental materials (slurry), which might have influenced the results of the study. The occurrence of DH is not only limited to the occlusal surface but also frequently in the cervical aspect of the teeth. The number, distribution, orientation, and diameter of the DTs are site-specific and the results may vary accordingly. Regular masticatory forces, eating habits of the individual, abrasiveness and pH of the food, and prophylactic procedures are among the many factors that could influence the outcome of the study. The extent of dentin collagen stability achieved by PA also needs to be specifically assessed. Further in vivo studies are required to validate the findings of this in vitro study.

## Conclusion

Within the limitations of this in vitro study, it can be concluded that all the remineralizing regimens occluded dentinal tubules, but to varying degrees. os-nHAp and PA/os-nHAp groups showed significantly superior TO, higher DoP, and lesser DP compared to CPP-ACP. PA pretreatment accentuated mineral deposition on demineralized dentin. CA challenge eroded only the surface mineralized layer in both os-nHAp and PA/os-nHAp groups; nevertheless, the tubular plugs, intratubular precipitates and DP remained unaffected compared to CPP-ACP.
